# Identification of Defective Maize Seeds Using Hyperspectral Imaging Combined with Deep Learning

**DOI:** 10.3390/foods12010144

**Published:** 2022-12-27

**Authors:** Peng Xu, Wenbin Sun, Kang Xu, Yunpeng Zhang, Qian Tan, Yiren Qing, Ranbing Yang

**Affiliations:** 1 College of Information and Communication Engineering, Hainan University, Haikou 570228, China; 2 College of Mechanical and Electrical Engineering, Hainan University, Haikou 570228, China

**Keywords:** hyperspectral imaging, maize seeds, defect detection, feature selection, convolutional neural network

## Abstract

Seed quality affects crop yield and the quality of agricultural products, and traditional identification methods are time-consuming, complex, and irreversibly destructive. This study aims to establish a fast, non-destructive, and effective approach for defect detection in maize seeds based on hyperspectral imaging (HSI) technology combined with deep learning. Raw spectra collected from maize seeds (200 each healthy and worm-eaten) were pre-processed using detrending (DE) and multiple scattering correction (MSC) to highlight the spectral differences between samples. A convolutional neural network architecture (CNN-FES) based on a feature selection mechanism was proposed according to the importance of wavelength in the target classification task. The results show that the subset of 24 feature wavelengths selected by the proposed CNN-FES can capture important feature information in the spectral data more effectively than the conventional successive projections algorithm (SPA) and competitive adaptive reweighted sampling (CARS) algorithms. In addition, a convolutional neural network architecture (CNN-ATM) based on an attentional classification mechanism was designed for one-dimensional spectral data classification and compared with three commonly used machine learning methods, linear discriminant analysis (LDA), random forest (RF), and support vector machine (SVM). The results show that the classification performance of the designed CNN-ATM on the full wavelength does not differ much from the above three methods, and the classification accuracy is above 90% on both the training and test sets. Meanwhile, the accuracy, sensitivity, and specificity of CNN-ATM based on feature wavelength modeling can reach up to 97.50%, 98.28%, and 96.77% at the highest, respectively. The study shows that hyperspectral imaging-based defect detection of maize seed is feasible and effective, and the proposed method has great potential for the processing and analysis of complex hyperspectral data.

## 1. Introduction

Maize (*Zea mays* L.) is one of the three major food crops in China, and it is also an essential feed and industrial raw material [1]. The quality of maize seed directly affects maize yield, food security, and the agricultural economy, making the issue of seed quality particularly important. In addition, with the development of modern agricultural machinery technology, especially the application of precision sowing machinery, the quality of seeds has been increasingly demanding [2]. Maize seeds are prone to damage, defects, and mildew during storage and transportation, where defects are significant indicators for seed quality evaluation [3]. Maize seeds of the same variety have many similar characteristics (such as color and shape) that make it difficult for human vision to distinguish them. Traditional detection methods (such as morphology, molecular biology, and genetic markers) are time-consuming, inefficient, and professionally demanding [4]. It can also cause irreversible damage to the sample, which is not conducive to a non-destructive and rapid evaluation of maize seed quality in industrial production [5]. In recent years, many scholars have applied machine vision, deep learning, and spectral sensing technology to seed quality detection, and it has achieved some research achievements [6]. Conventional machine vision techniques can detect severe external defects in seeds. However, when pest symptoms are not obvious, internal information about the sample cannot be obtained for identification, resulting in poorer detection accuracy. Hyperspectral imaging (HSI) is an effective non-destructive inspection technique that combines traditional spectral information (reflecting chemical composition) with image information (reflecting physical properties) [7]. It can also be used to solve the problem that the defective areas of the seeds are obscured during the detection process. The subdivided wavelength data in hyperspectral has a unique role in reflecting subtle changes in the intrinsic physiological properties of seeds, such as moisture, starch, protein, and fat [8]. Therefore, the most sensitive hyperspectral characteristic parameters of different quality seeds can be obtained by analyzing the spectral response of seed wavelengths.

Sellami proposed a new hyperspectral image classification method based on a multi-view deep neural network that fuses spectral and spatial features using only a small number of labeled samples [9]. Alimohammadi successfully identified and classified three varieties of maize seeds using hyperspectral nondestructive imaging in the wavelength range of 400–1000 nm [10]. Zhang developed a new method for identifying different varieties of commercial maize seeds using visible and hyperspectral imaging techniques in the near-infrared band to develop a new method for identifying different varieties of commercial maize seeds [11]. Liu proposed a method for identifying the purity of hyperspectral rice seeds based on the LASSO logistic regression model by combining the advantages of the sparsity property of the least absolute shrinkage selection operator (LASSO) algorithm and the classification property of the logistic regression model (LRM) [12]. Due to the complexity of hyperspectral data, not all spectral variables are related to the target components, and the full spectrum inevitably contains a large amount of noise, invalid information, and even interfering variables [13]. This situation increases the complexity of the model but also seriously affects its accuracy and reliability. Therefore, feature selection has become a critical step in the hyperspectral modeling process, which can quickly obtain the optimal subset of feature wavelengths [12].

Feature selection has been a hot research topic in recent years in hyperspectral analysis techniques and chemometrics, where the more common method is the traditional spectral feature selection algorithm [14]. Nagasubramanian used a genetic algorithm (GA) as an optimizer for optimal band selection, and the accuracy of identifying charcoal rot infection in soybean with the selected combination of six bands reached 97% [15]. Pang used successive projections algorithm (SPA) to select 25 feature variables, which could achieve 99.77% accuracy in predicting Quercus variabilis seed vigor [16]. Song used competitive adaptive reweighted sampling (CARS) to screen 60 sensitive wavelengths, in which the root mean square error (RMSE) and calibration set accuracy of the diagnostic model were 1.97 and 0.87, respectively, which could accurately predict chlorophyll content in maize canopy and provide a reference for rational use of fertilizer [17]. Zhang showed that the CARS algorithm (29 wavelengths) outperformed the SPA algorithm (24 wavelengths) in feature variable selection and achieved an accuracy of 89% in the validation set based on the deep forest (DF) model, which can effectively identify the sound or slightly sprouted wheat kernels (slightly sprouted wheat kernels) [18]. However, the above traditional feature selection methods utilize only spectral data without considering the labels in the dataset, which may lead to a low relevance of the selected feature subset to the final target. With the rapid development of deep learning techniques, convolutional neural network (CNN)-based methods have been successfully applied to hyperspectral band selection [19]. Yuan proposed a point-centered convolutional neural network incorporating embedded feature selection for feature selection, extraction, and classification, and the accuracy of the selected five critical bands reached 97.98% for non-destructive and rapid identification of moldy peanuts [20]. Sharma proposed a CNN named DeepFeature applied to non-image data for feature selection with 98% classification accuracy on an independent test set, which can provide a powerful method for identifying biologically relevant gene sets [21].

With the development and research of artificial intelligence, the application of deep learning combined with HSI technology to agricultural tasks has gradually become a research hotspot where more and more researchers are using deep learning models represented by CNN to solve the problem of seed quality detection [6,22]. Yu achieved the recognition of hybrid okra seeds using HSI (948.17–1649.20 nm) technology, and its recognition rate reached 97.68%, demonstrating that the CNN model has reliable advantages in achieving high accuracy and stability [23]. Pang showed that building a CNN model based on spectral images (497.72–998.16 nm) has excellent results for identifying the seed viability of Sophora japonica, which is significant for the study of seed vigor and spectral change mechanisms [24]. Gao used a one-dimensional convolutional neural network (1D-CNN) to classify aflatoxins in maize and peanuts based on hyperspectral data (292–865 nm), and the classification accuracy was 96.35% and 92.11%, respectively, which remarkably improved the detection efficiency [25]. Li proposed a method based on a deep convolutional generative adversarial network (DCGAN) and near-infrared hyperspectral imaging technique (866.4–1701.0 nm) for identifying unsound wheat seeds, and the accuracy could reach up to 96.67% [26]. The above shows that the joint model based on DCGAN and CNN has good performance for identifying small samples and can learn more feature information from spectral data. In order to further optimize the deep learning model, some scholars have introduced the attention mechanism to focus on critical information selectively [27,28]. Of these, Wang developed an attention-based CNN approach (Geo-CBAM-CNN) for crop classification using time-series Sentinel-2 images. The results show that the convolutional block attention module (CBAM) can help mitigate the effect of geographical heterogeneity and suppress unnecessary information [28]. The proposed model achieves an accuracy of 97.82%, demonstrating its superior performance in large-scale applications. In summary, CNN is an accurate identification model, and combining it with HSI can improve the overall model performance by using the internal and external feature information of samples. Therefore, it is important to establish an identification model for maize seed defects using CNN combined with HSI and to explore the effects of different feature selection methods and classification models on the identification results.

This study investigates the feasibility of HSI combined with CNN for the identification of maize seed defects. The objectives of this study are as follows: (1) to collect image information of defective maize seeds, extract spectral data of the region of interest, and preprocess them to analyze the differences between defective and healthy maize seeds; (2) to propose a CNN model (CNN-FES) based on feature selection mechanism for selecting key feature wavelength variables that are beneficial to the target task, and to evaluate the impact of the variables selected by different feature selection methods on the model; (3) to design a CNN model (CNN-ATM) based on attentional classification mechanism in order to discriminate the samples, and the best model for the identification of maize seed defects was selected by comparing the classification results of different machine learning methods (LDA, RF, and SVM) and CNN-ATM.

## 2. Materials and Methods

### 2.1. Sample Preparation and Hyperspectral Image Acquisition

**Figure 1:**
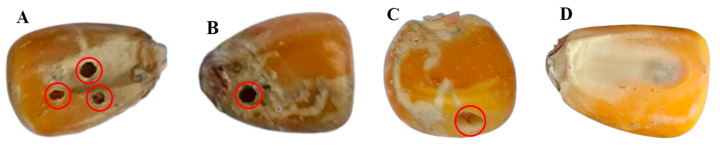
Images of maize seed samples: (**A**) embryonic surface worm-eaten; (**B**) non-embryonic surface worm-eaten; (**C**) lateral surface worm-eaten; (**D**) healthy.

Maize seeds of “Zhengdan 958” collected in 2022 from the local farmers’ market in Haikou City (20.06°N, 110.33°E), Hainan Province, were used as material for this study; this variety is widely grown in China. All samples were identified and selected by agricultural cultivation experts, and they were analyzed in three aspects: morphology, structure (with endosperm), and end-use type (cultivation). From the samples, 200 worm-eaten seeds (Figure 1A–C) and another 200 healthy seeds (Figure 1D) were chosen as the control group, numbered in order, and placed in sealed bags. In order to eliminate the interference of moisture during spectral data collection, all samples were stored at room temperature (26 °C) for 12 h. These maize seeds did not perform other physical or chemical treatments before acquiring spectral information, and the shapes and colors between them were roughly the same. In this study, the division of the training set and test set was carried out randomly in the ratio of 7:3, with 280 samples in the training set and 120 samples in the test set.

**Figure 2:**
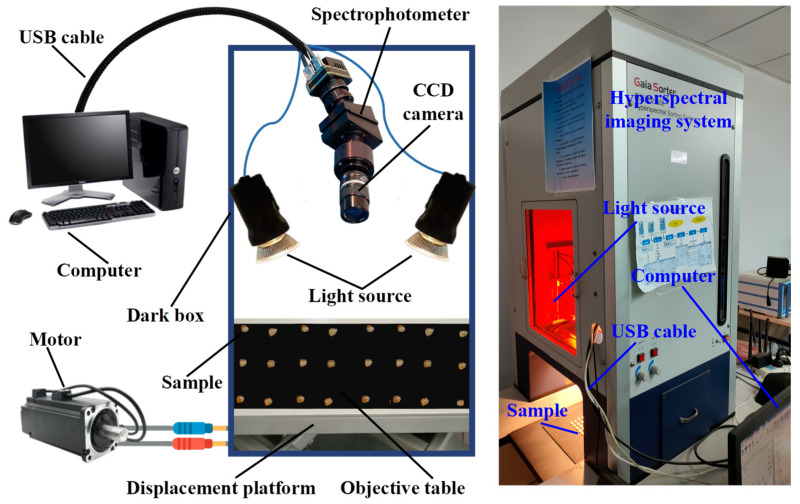
Hyperspectral image acquisition system.

As shown in Figure 2, the hyperspectral image acquisition system is the “GaiaSorter” hyperspectral sorting equipment manufactured by Zolix Co., Ltd. (Beijing, China). The whole system consists of five parts: a uniform light source (2900-ER+9596-E, Illumination, East Syracuse, NY, USA), a “Image−λ” series of spectral cameras (Imspector N17E; TE-cooled InGaAs CCD, Spectral Imaging Ltd., Oulu, Finland), an electronically controlled mobile platform (MTS 120, Beijing optical instrument factory, Beijing, China), a computer (OptiPlex 7090, Dell, Round Rock, TX, USA), and control software (SpecView Ltd., Uckfield, UK). The system has a spectral acquisition range of 866.4–1701 nm and contains 254 bands, and a three-dimensional data cube containing image information and spectral information of all maize seed samples can be obtained by one scan at a time. In addition, the system includes a dark box to isolate the interference of external light or noise and an object stage to place the seed samples.

The equipment was allowed to warm up for 0.5 h before capturing the spectral images, and then the camera exposure time was tuned and determined to be 30 ms. The distance between the camera and the sample was adjusted to 36 cm, the image resolution at full frame was 1392 × 1040, and the movement speed of the mobile platform was 2 mm/s. The maize seeds were placed single on the blackboard of the object stage in 7 rows and 6 columns. The computer-controlled mobile platform was moved from left to right, and the camera gathered spectral images of the samples, which were then transferred to the computer for storage. Due to the inhomogeneous intensity distribution of the light source and the presence of dark current noise, black and white correction of the original hyperspectral images is required to improve the signal-to-noise ratio of the acquired images [1]. First, we placed a whiteboard for white reference to obtain a corrected image with a reflectivity of about 100%. Then, we placed a blackboard for black reference to obtain a corrected image with a reflectivity of 0. Finally, we calculated the relative reflectivity of the calibrated image according to Formula (1) [29].

(1) $$R_{e}=\frac{I_{r}-I_{d}}{I_{w}-I_{d}}$$

where $R_{e}$ is the relative reflectivity of the calibrated image, $I_{r}$ is the spectral reflectivity of the original image, $I_{w}$ is the spectral reflectivity of the corrected whiteboard, and $I_{d}$ is the spectral reflectivity of the corrected blackboard.

### 2.2. Spectral Data Extraction and Preprocessing

**Figure 3:**
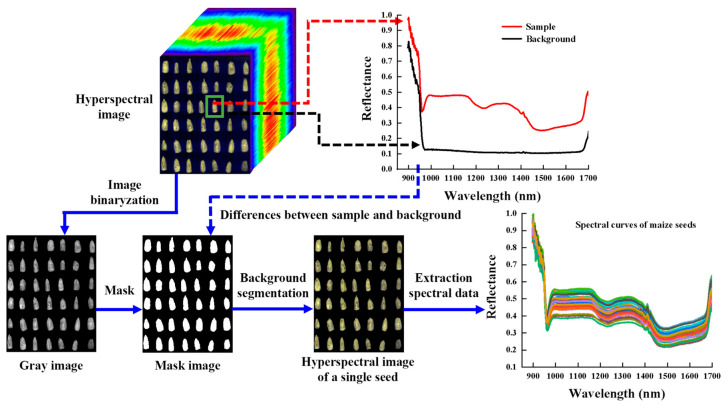
The process of hyperspectral image segmentation and spectral extraction.

In order to obtain the representative spectral information of the samples, the data processing of the original hyperspectral images is required, and the implementation process is shown in Figure 3. First, the background and noise of the original hyperspectral image were removed using Matlab R2020a (MathWorks, Natick, Massachusetts, MA, USA) combined with the morphological filtering method, which was converted to a grayscale image by threshold segmentation, and the single seed region was retained as the region of interest (ROI) of the image. Next, the grayscale image was converted into a mask image based on the difference between the spectral reflectance values of the sample and the background, and the hyperspectral image of the ROI was separated from the background by multiplying the original hyperspectral image with the mask pixel points to obtain the spectral reflectance image of each seed. Last, the average value of all pixel points (per band) within the ROI was extracted as the average spectrum of the sample using ENVI 5.3.

The experiments are disturbed by external factors such as the measurement state of maize seeds, the working state of the equipment, and the environment, which lead to random noise and baseline drift in the obtained spectral data, which can have a significant impact on the results of spectral data analysis. It is necessary to pre-process the spectral data to highlight the valid information of the spectral to reduce the influence of these conditions on the robustness of the model and improve its prediction accuracy. This study uses Matlab R2020a to denoise the spectral data, and after the Savitzky-Golay (SG) processing [30], the DE and MSC methods were used to pre-process the data and compare these two methods, respectively [31]. Among them, the SG can improve the smoothness of the spectra and reduce noise interference, and a smoothing window of 11 and a polynomial number of 1 are selected to process it in this paper. DE can improve the baseline offset phenomenon and focus the analysis on the fluctuation of the data trend itself. MSC can improve the signal-to-noise ratio of the spectral, effectively eliminate the effect of scattering, and enhance information on the spectral absorption related to the component content.

### 2.3. Traditional Feature Selection and Machine Learning Methods

The wavelength range of the original spectra was 866.4–1701.0 nm (254 bands). In order to remove noise interference caused by factors such as equipment and environment, the wavelength range of 897.4–1701 nm (245 bands) was selected for analysis in this study. In practical applications, the amount of spectral data is usually enormous. Using spectral data at the full wavelength will increase the complexity of model calculations, and its modeling effects will be affected by redundant information and collinearity problems. Therefore, to further investigate the feasibility of applying hyperspectral techniques to identify defects in maize seeds, first, conventional SPA and CARS were used for feature selection to reduce the amount of modeling data and ensure its identification on the model, both of which were implemented using Matlab R2020a. Second, traditional machine learning algorithms, including LDA, RF, and SVM, were used to establish a hyperspectral classification model to identify healthy and defective maize seeds. These three methods are all implemented using Python’s sklearn toolkit (https://scikit-learn.org/stable/ (accessed on 13 November 2022).

#### 2.3.1. Feature Selection Method

SPA is a method that minimizes the overlap of spectral information, using vector projection to select effective feature wavelengths that have low redundancy yet reflect the critical information of the sample spectrum [32]. The algorithm can eliminate collinearity between the combinations of feature variables through recursive computation to select new wavelengths, thus improving the conditions of classification and regression tasks [33]. It can also remove spectral regions with high noise and irrelevant information to achieve screening of sensitive variables and improve crop identification [34]. In addition, CARS is an alternative approach to feature selection that allows assessing the importance of each variable based on the absolute value of the regression coefficients of the partial least squares model [35]. Monte Carlo resampling was performed iteratively and competitively to make the distribution of the selected band positions more uniform [36]. Different subsets were evaluated using cross-validation, and the selected best variables combination is more suitable for subsequent modeling [37].

#### 2.3.2. Machine Learning Method

LDA is a powerful supervised learning technique that can significantly increase the discrimination ability between classes based on the distance between projections and effectively classify data [38]. LDA pays more attention to the inter-class distance and intra-class distance of the projected samples in the new dimension space, ensuring that the model has the best separability in the subspace [39]. The method used in this study to solve the LDA hyperplane eigenmatrix is singular value decomposition (SVD), and the threshold used for rank estimation in the SVD solver is 1 × 10^−4^.

SVM is designed based on statistical principles and follows the structural risk minimization principle to obtain stable classification results by maximizing the decision boundary [40]. The radial basis function (RBF) is used to transform nonlinear problems into linear ones [41]. The optimal values of its penalty coefficient C and kernel function gamma are determined by the grid search method, and their search range is set to 10^−5^–10^5^. This study uses a 10-fold cross-validation strategy to set C and gamma to be 10 and 0.004, respectively.

RF is an integrated learning method that can be used for classification, combining the results of decision tree-based modeling and obtaining the final estimation results by voting [42]. The grid search method seeks the appropriate number of decision trees (n_estimators), the number of randomly selected variables at nodes (random_state), and the maximum number of features (max_features) to correct the overfitting due to the inductive preference of decision trees [41]. This study uses the Gini coefficient as a measure to determine n_estimators, random_state, and max_features to be 15, 2, and 6, respectively.

### 2.4. Convolutional Neural Network Architecture for Feature Selection and Classification

**Figure 4:**
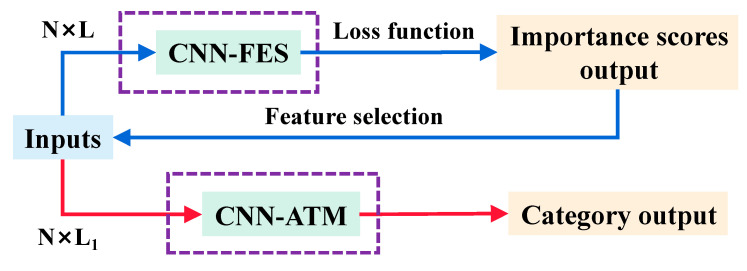
CNN network processing flow.

CNN is a deep learning method with structural diversity and nonlinear transformation that has achieved significant achievements in many fields such as image processing, speech recognition, and text data [20,24]. In recent years, CNN has been intensively researched and explored in spectral analysis and extended to applications for one-dimensional (1D) data (such as pixel-level spectra) and three-dimensional (3D) data [43]. This study proposed a novel 1D-CNN model for hyperspectral analysis, in which the feature shape of the spectral data of the sample is 1 × 245. The model consists mainly of two parts. One is a CNN architecture based on a feature selection function for optimal wavelength selection; the other is a CNN architecture based on an attention mechanism for identifying defective seeds. We define them as CNN-FES and CNN-ATM. These two custom modules were annotated through purple dashed boxes, and the processing process of the whole network is shown in Figure 4. First, the input data of shape N × L (N is the number of samples, and L is the number of features) was processed by the feature selection module, which evaluates the weight of the coefficients in the network iteration through the loss function, according to the output importance score (IS) selects the input. Then, the selected combination of feature variables (N × L_1_) was input to the convolutional classification module for processing, and healthy and defective seeds were identified based on category output.

#### 2.4.1. CNN Architecture Based on the Feature Selection Mechanism

**Figure 5:**
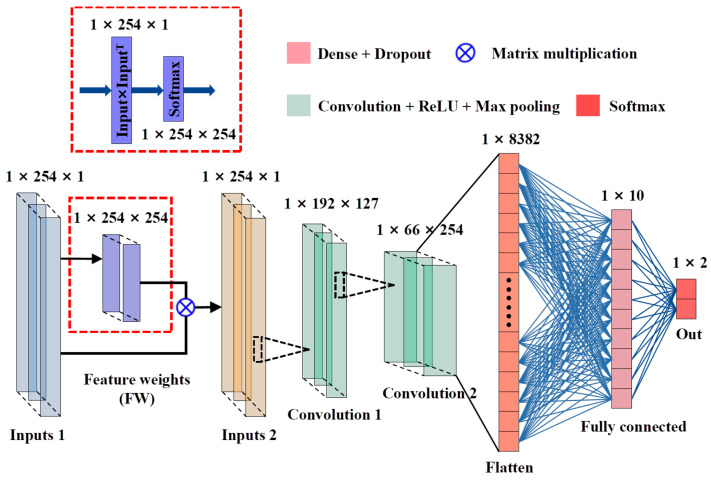
Architecture of CNN-FES module.

For the CNN-FES module, the architecture is shown in Figure 5. The feature selection mechanism allows the network to select certain vital variables of the input data while ignoring the selection of unimportant variables. Referring to the Vaswani algorithm, we calculate the value of the loss function in the CNN network to indicate the importance of each feature in the target classification task and select the feature wavelengths based on IS. The feature weight (FW) block was annotated with a red dotted box, and the weight of FW is output in the same shape as the input.

Figure 5 shows the processing of the CNN-FES module. First, the input data was processed by the nonlinear activation function Softmax after the FW operation, and then the output weight coefficients were multiplied by the input. The weighted vector of the FW block is defined as the output and calculated as shown in Equation (2) for the subsequent convolution operation and the calculation of IS.

(2) $$Y_{FW}=f_{S}\left(W_{FW}\right)*X$$

where $X=\left[X_{1},X_{2},\ldots .,X_{L}\right]$, $W_{FW}=X*X^{T}$, $X^{T}$ denotes the transpose matrix of X and ∗ denotes the multiplication of the corresponding elements in the two matrices. $W_{FW}$ is the inner product of the vector, $Y_{FW}$ is the weighted vector of the FW block, $f_{S}$ is the activation function, and $f_{S}\left(W_{FW}\right)$ is the weight coefficient. The geometric meaning of $W_{FW}$ is the angle between two vectors, which is the projection of one vector onto another vector. The larger the value of $W_{FW}$, the higher the correlation between the two vectors, thus giving them more attention in the feature extraction of convolution operation, and the IS will be higher. In addition, alternatives to $f_{S}$ are Sigmoid, Tanh, and ReLU. Sigmoid makes the output not centered on 0, which will reduce the efficiency of weight update. Tanh will make part of the input negative, which is not conducive to model training. ReLU sets part of the output to 0 and deletes related feature information. The denominator of Softmax combines all the factors of the original output value, making the outputs associated with each other. Therefore, Softmax was selected in this study to output the weight coefficients.

Next, the $Y_{FW}$ output from the FW block was reconstructed into N × L × 1 for convolutional operation, and two 1D convolutions (Convolution 1/Convolution 2) blocks (number of kernels, kernel size, and strides were set to 127/245, 64/32, and 1/1, respectively), one flatten layer, and one dense layer (number of neurons is 10) were processed, and the last is the output layer (Softmax). Of them, the convolutional layers use the ReLU activation function with L2 regularization added. The MaxPooling layer (pool size and strides of 2, and 1, respectively) was downsampled after each convolutional layer, and the dropout layer (rate is 0.25) was performed after the dense layer.

In the feature selection process, features unrelated to labels or random noise will be related through model training, resulting in the neglect of a lot of potentially valuable information. Referring to the idea of the Altmann algorithm [44], “permutation importance” was used for processing in this study. We disordered the labels five times, then obtained the feature importance (IF) under the false labels, selected the features based on the difference between the IF under the true and false labels, and defined the IS calculation as shown in Formula (3).

(3) $$IS=\log\left[\left(1e-10+S1_{k}\right)/\left(S2_{k}\right)\right]$$

where k denotes the kth characteristic variable (k = 1, 2, …, 245), $S1_{k}$ denotes the IF of variable k before no shuffle, and $S2_{k}$ denotes the 75% quantile of the IF of all variables k after multiple shuffles. IF is the loss value calculated by the model at each iteration based on the loss function ($Loss_{yp}$); 1e−10 and 1 are smoothing methods used to avoid numerator and denominator zeros during computation, and they have no practical significance. If the value of *IS* < 0, indicates that the feature variable k is not a vital feature of the classification task.

Finally, the Adam optimization algorithm was used to train the feature selection model to minimize the classification error while retaining a minimum $f_{S}\left(W_{FW}\right)$ value, and the defined loss function is ${\mathrm{Loss}}_{\mathrm{yp}}$ shown in Formula (4).

(4) $$Loss_{yp}=-\frac{1}{N}\overset{N}{\underset{i=1}{\sum }}\overset{M}{\underset{j=1}{\sum }}y_{ij}\log\left(p_{ij}\right)+\lambda \overset{M}{\underset{j=1}{\sum }}\left(softmax\left(w_{j}^{2}\right)\right)$$

N indicates the number of samples, M indicates the number of categories, $y_{ij}$ indicates the label value, $p_{ij}$ indicates the predicted value, $w_{j}$ indicates the weight vector, and λ indicates the regularization coefficient. $Loss_{yp}$ consists of two parts: one is the cross-entropy loss, which controls the classification accuracy of the target task; the other is the sum of the weight coefficients in the FW block, which makes the absolute value of the weights tend to decrease overall and the IS of the features can take full advantage of all the inputs from the upper layer.

#### 2.4.2. CNN Architecture Based on Attention Classification Mechanism

**Figure 6:**
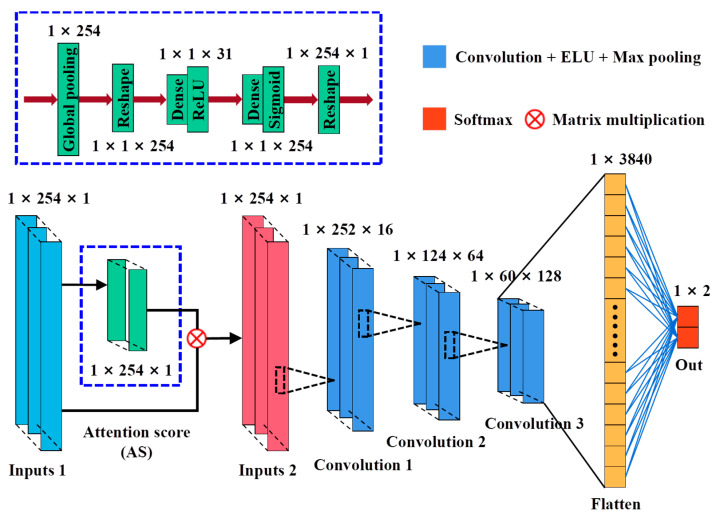
Architecture of CNN-ATM module.

For the CNN-ATM module, the architecture is shown in Figure 6. The attention classification mechanism makes the network focus more on the information in the input data that is more critical to the current task and reduces the attention to other non-critical information. By referring to the SeNet algorithm [45], the importance degree (ID) of the feature channel was obtained through network learning, and then enhances the beneficial features and suppresses the features that are useless for the current according to the ID, and uses it to achieve the identification of defective maize seeds. The attention score (AS) block was annotated with a blue dashed box, and the weights of AS are output in the same shape as the input.

Figure 6 illustrates the processing of the CNN-ATM module. First, the input data was reshaped after global average pooling, and the weights of each feature channel were obtained using the nonlinear activation function Sigmoid after the two fully connected, and the weights of the output were reshaped again and multiplied with the input. The weighted vector of AS block was defined as the output, and the computation was performed according to the following Formula (5) for subsequent convolution operation and ID calculation.

(5) $$Y_{AS}=f_{S}\left(W_{2}*f_{R}\left(W_{1Z}\right)\right)*X$$

where, $X=\left[X_{1},X_{2},\ldots .,X_{L}\right]$, ∗ represents the multiplication of corresponding elements in two matrices, the dimension of $W_{1Z}$ is 1 × 1 × L/r, r is the scaling parameter, which was taken as 8 in this study, the dimension of $W_{2}$ is L × L/r, $Y_{AS}$ is the weighting vector of the AS block, $f_{S}$ and $f_{R}$ are the activation functions, and $f_{S}\left(W_{2}*f_{R}\left(W_{1Z}\right)\right)$ was defined as the weight coefficient.

Second, the output ($Y_{AS}$) from the AS block continued to complete the convolution operation and performed three 1D convolutions (Convolution 1/Convolution 2/Convolution 3) blocks (the number of kernels, kernel size, and strides were set to 16/64/128, 3/3/3, and 1/1/1, respectively), and one flatten layer, and the last part is output layer (Softmax). Where the ELU activation function was used for the convolutional layers, and L2 regularization was added. The downsampling operation of the MaxPooling layer (pool size and strides of 2, and 1, respectively) was performed after each convolutional layer. Finally, the Adam optimization algorithm was used to train the classification model to obtain the best detection effect of defective seeds.

### 2.5. Model Training Process and Evaluation Metric

In this study, the training set (280) was used for model training and the test set (120) was used for the performance evaluation of the model. The sample features were normalized (z-score) to compensate for scale differences between the data before they were fed into these classification models. In addition, the obtained spectral data were statistically analyzed using IBM SPSS 26 using paired sample t-tests to assess statistical differences in the spectral characteristics of the samples. The mean, standard deviation, and standard error mean of the samples were calculated by mean comparison analysis to analyze if significant differences were presented between healthy and defective maize seeds. The remaining parameters of the models for LDA, SVM, and RF were set as default, and then their classification results were compared with the CNN models. We optimize the hyperparameters of both CNN-FES and CNN-ATM models by analyzing the accuracy of the training and test sets on epoch to obtain the output value of the minimized loss function and the model with good robustness. The number of epochs on CNN-FES was set at 100, the batch size was 4, λ was 0.15, and the weight decay value was 0.1. The number of epochs on CNN-ATM was 200, the batch size was 32, and the weight decay value was 0.008. The initial value of the learning rate for both was 0.001, the momentum value of the gradient descent optimizer was set to 0.9, and the learning rate decays automatically according to the number of iterations. The above CNN model was built and implemented using the programming language Python 3.8 (https://www.python.org/ (accessed on 13 November 2022)) in the machine learning platform TensorFlow 2.3 (https://devdocs.io/tensorflow~2.3/ (accessed on 13 November 2022).) and the deep learning framework Keras 2.4.3 (https://keras.io/ (accessed on 13 November 2022)), using an AMD Ryzen 7 3800 × 8-core processor, 3.90 GHz 16 GB, and NVIDIA GeForce RT × 3050 graphics processing unit for training and testing.

For the evaluation strategy, this study uses the standard metrics of the classification task to calculate the accuracy ($A_{C}$), sensitivity ($S_{E}$), and specificity ($S_{P}$) of the model based on the confusion matrix using Formulas (6)–(8), respectively, to measure the ability of the model to discriminate the samples.

(6) $$A_{C}=\frac{TP+TN}{TP+FP+TN+FN}$$

(7) $$S_{E}=\frac{TP}{TP+FN}$$

(8) $$S_{P}=\frac{TN}{TN+FP}$$

In this case, *TP*, *TN*, *FP*, and *FN* represent true positive, true negative, false positive, and false negative of the confusion matrix, respectively.

## 3. Results and Discussion

### 3.1. Spectral Data Analysis

**Figure 7:**
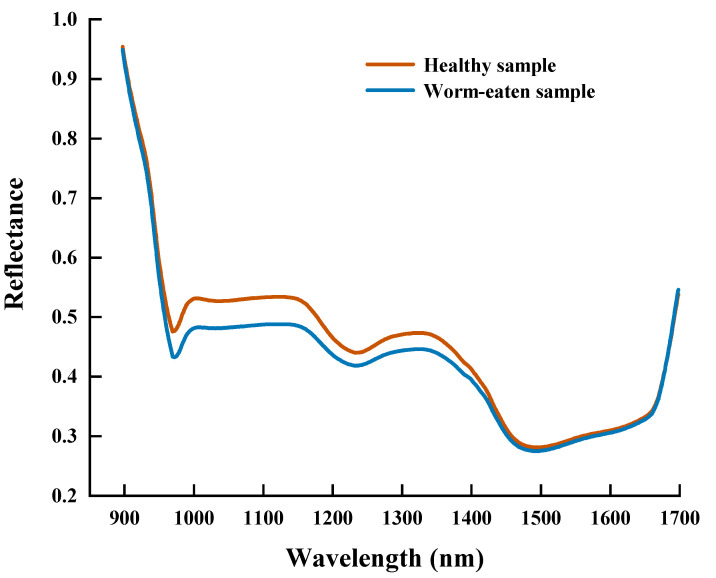
The average spectral curve of maize seeds.

This study analyzed the differences in spectral data using paired-sample t-tests. Among them, the mean spectral value of healthy maize seeds (0.448) was significantly higher than that of defective ones (0.426), and they showed a significant difference (t = 21.259, *p* = 0.001 < 0.05). The large difference in the spectral characteristics data between the two implies that the presence of defects in maize seeds leads to a change in their internal structure. This change affects the magnitude of its spectral reflectance and can better reflect the actual state (healthy or defective) of the maize seeds. The average spectral information of the maize seed samples in the wavelength range of 897.4–1701 nm is shown in Figure 7. It can be seen that the general trend and characteristic peaks of the spectral curves of healthy and worm-eaten seeds are the same, with the spectral curves showing a significant decrease in the wavelength range of 897.4–959.3 nm and a leveling off in the wavelength range of 993.4–1666.4 nm. Although some regions overlap, the reflectance of different wavelengths is slightly different, and the spectral data can be further studied and analyzed. The spectral reflectance of the defective seeds at 952.4–1484.5 nm was lower than that of healthy maize seeds due to changes in their protein, starch, and moisture composition. Among them, the average spectral curves show absorption peaks or valleys at 959.3 nm, 996.9 nm, 1159.1 nm, 1239.1 nm, and 1331.5 nm, which are more affected by the damage to the structure and tissues inside the maize seeds. Traditional visual detection methods are based on surface defects of seeds in visible light, but most of the spectral signals are invisible to the human eye, which can provide a great deal of information about the internal defects of seeds.

The troughs around 980 nm were caused by the joint action of stretching vibrations of N-H in protein and C-O and C-N in soluble sugar, and the absorption peak near 1000 nm is related to the third overtone of N-H stretching in proteins [46]. There are two noticeable troughs around 1235 and 1500 nm, where the former represents the absorption wavelength of the second overtone of C-H stretching in carbon hydrate, and the latter represents the absorption wavelength of the first overtone of O-H stretching in water and N-H stretching in protein, respectively [23]. The absorption peak near 1350 nm was correlated with the second overtone of C-H stretching in starch, and it is also the absorption region of C-O stretching vibration, and the valley of absorption around 1660 nm is related to the fat content [47]. These characteristic peaks reflect the degree of absorption of different wavelengths of near-infrared light by the high molecular compound in maize seeds. In addition, worm-eaten seeds lost some of their original higher structures, resulting in broken chemical bonds and disruption of the molecular structure of substances such as proteins, starch, and water, which led to differences in reflectance on the spectral curves [2]. These differences demonstrate the distinction between healthy and worm-eaten maize seeds for one thing and the feasibility of HSI for defect detection of maize seeds for another.

**Figure 8:**
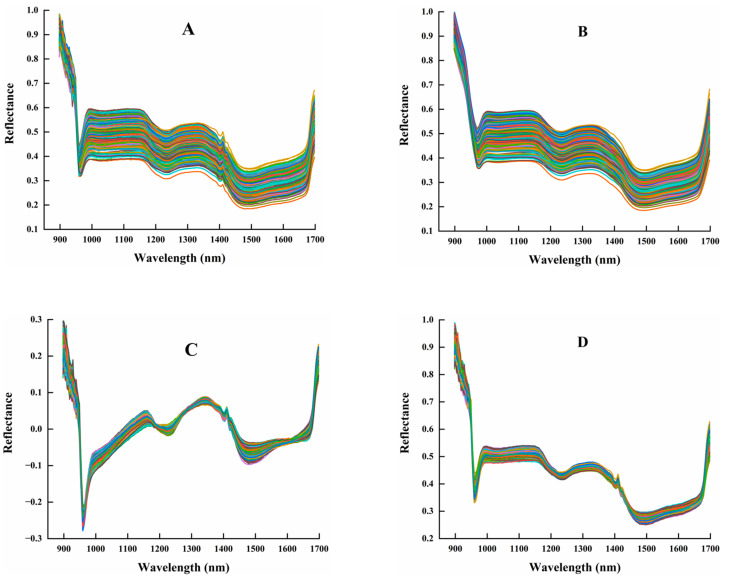
Spectral curves of maize seed samples under preprocessing conditions: (**A**) Raw; (**B**) SG; (**C**) DE; (**D**) MSC.

Figure 8B shows the spectral curves of the samples after SG pretreatment, from which it can be shown that the SG method retains almost all the information of the original spectral curves, and the modeling results after the preprocessing may not differ much from the modeling results using the original spectra directly. Figure 8C,D show the spectral curves of the samples pretreated by detrending (DE) and multiple scattering correction (MSC), respectively, from which it can be seen that the DE method changes the overall trend of the original spectral, while the MSC method retains the overall trend of the original spectral. However, it can be found that both of them enhanced the characteristics of the spectral absorption curves and decreased the discreteness of the curve after pretreatment, and the use of these two preprocessing methods can effectively reduce noise interference in the spectral data.

### 3.2. Results of Feature Wavelength Selection

**Figure 9:**
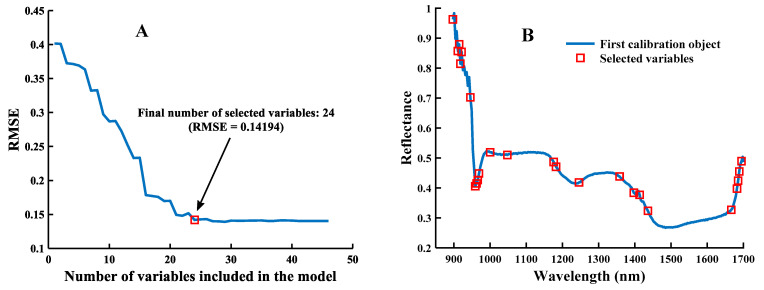
SPA selected feature wavelengths: (**A**) the value of RMSE varies with the number of variables; (**B**) the selected wavelength variables.

Set the selection range of the characteristic wavelength of SPA to 1–50, and the result is shown in Figure 9, taking the spectral data processed by MSC as an example. In this study, the RMSE value was used as the main evaluation index to screen the best combination of variables, and a total of 24 characteristic variables were obtained at the minimum RMSE value of 0.14194 (the coefficient of determination R^2^ of the model is 0.9864), accounting for 9.80% of the total wavelengths. From Figure 9A, it can be seen that the RMSE values showed an overall decreasing trend when the number of variables was less than 24, and the changing trends tended to be gentle when the number of variables was greater than 24. Figure 9B indicates the selection of specific variables, and the red “☐” represents the selected variables with the smallest RMSE value corresponding to the optimal number of characteristic wavelengths. The above indicates that the selected characteristic wavelengths contained information about worm-eaten maize seeds and had higher discrimination than healthy ones. Therefore, the reflectance values corresponding to the 28 wavelengths were selected as the data for subsequent modeling.

**Figure 10:**
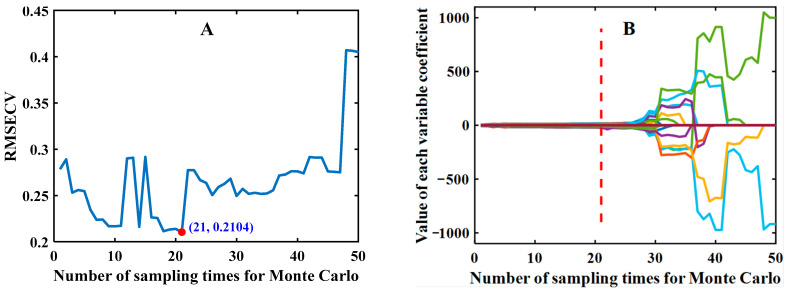
CARS selected feature wavelengths: (**A**) the value of RMSECV varies with the number of sampling; (**B**) the value of the variable’s coefficient.

The results selected by CARS (set the number of Monte Carlo sampling N to 50 and 5-fold cross-validation) are shown in Figure 10, taking the spectral data processed by DE as an example. As can be seen in Figure 10A, the subset of spectral data changed at each sampling, resulting in a trend of decreasing and then increasing root mean square error of cross-validation (RMSECV) values. The above indicates that at the initial stage of sampling (0–21), a large amount of information irrelevant to identifying defective maize seeds or partially co-linear information was removed, and the RMSECV achieved a minimum value (0.2104) when the sampling number was 21. With the sampling number increased, and the removal of critical information from the spectral data, the model performance becomes progressively worse. Figure 10B shows the trend of the regression coefficient of each wavelength variable in the process of variable optimization, and the position marked by the red dashed vertical line is the minimum value of RMSECV, at which the subset of spectral data obtained by sampling is the optimal wavelength combination, containing 34 wavelength variables, accounting for 13.39% of the total wavelengths.

**Figure 11:**
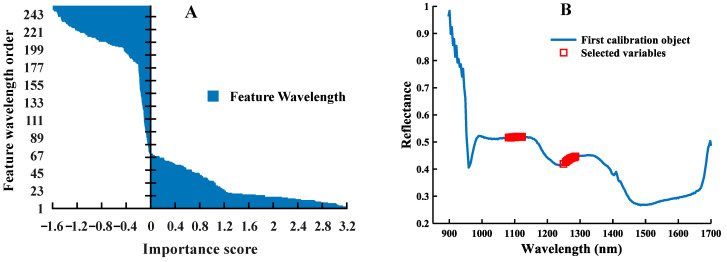
CNN-FES network selected feature wavelengths: (**A**) the value of IS of each feature wavelength; (**B**) the selected wavelength variables.

The result of the feature selection of the CNN-FES network proposed in this study is shown in Figure 11 (taking the spectral data processed by MSC as an example), and its score is of great significance for the object classification task. Figure 11A shows the IS at each wavelength obtained by inputting the raw hyperspectral data into the CNN-FES network. Since this method shows good selectivity, the IS value of some unimportant wavelengths will be less than 0, so 24 wavelength variables with higher values (the first 10%) can be selected (which can be adjusted according to the needs of practical applications), and the detailed distribution of its selection results on the spectral curve is shown in Figure 11B. It can be seen that there are more continuous wavelengths in the selected subset of effective wavelengths (the red “☐” represents selected variables). This situation indicates that the correlation between adjacent feature wavelengths is high, and the selected subset has less redundant information, which may be more favorable for the model to achieve better classification results. In addition, the selected feature wavelengths are all at and near the wave peaks on the average spectral curve, indicating that the wavelength selection method based on the CNN-FES model proposed in this study can effectively capture important feature information in the spectral data. Moreover, this phenomenon can be explained by the difference caused by the stretching vibration caused by the absorption of chemical functional groups in the biochemical components of maize seeds, indicating that our proposed selection method is representative and interpretable, which can be used to solve practical agricultural problems.

Feature selection is one of the important methods for data dimensionality reduction, and it has been a hot topic of research in the field of spectral data analysis. Therefore, in this study, the proposed CNN-FES method was compared with two traditional SPA and CARS feature selection methods, and the results of feature wavelengths selected from the preprocessed spectral data (245 variables) are shown in Table 1. As can be seen, the feature wavelengths selected by these algorithms are very different. This case is because they use different principles, with SPA focusing on the comparison of projection vector sizes, CARS tending to use thresholds to control the number of variables selected, and CNN-FES focusing on the interactions and differences between variables.

### 3.3. Analysis of Modeling Results

#### 3.3.1. Detection Results Based on Full Wavelength

**Figure 12:**
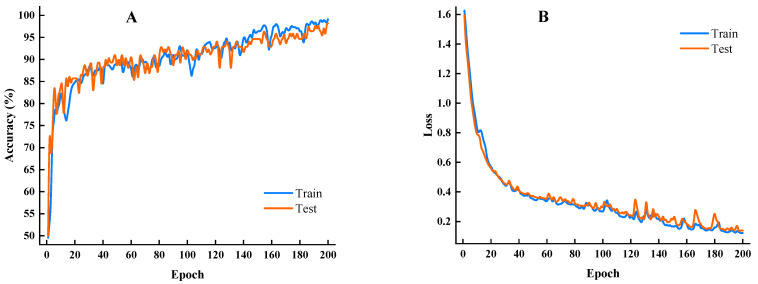
The training results of the CNN-ATM network: (**A**) classification accuracy of the model; (**B**) loss of the model.

In order to compare the discrimination results of different models, this study inputs the spectral data preprocessed by DE and MSC into the LDA, RF, and SVM models and the proposed CNN-ATM network for modeling, respectively. Figure 12A,B show the accuracy curve and loss function curve of CNN-ATM training, respectively, which can observe the training process and classification effect in real-time. The proposed model achieved an accuracy of 85% at 20 epochs, and it improved rapidly as the number of epochs increased. The loss dropped to 0.3 in the 100th epoch, and it continued to decrease around this low loss. This result indicates that the model has a good convergence speed and generalization. After 200 iterations, the model achieved its best performance and showed higher stability in classification tasks.

The detection results of the four models on the full wavelength were compared in this study, and their accuracy in the training set and test set is shown in Table 2. The classification accuracy of all models was above 91%. The RF model showed the worst results, and its $A_{C}$ (91.67%), $S_{E}$ (87.50%), and $S_{P}$ (96.43%) on the test set were lower than those of the other models. It can be seen that the spectral data preprocessed by DE has a better classification effect than those preprocessed by MSC. The $A_{C}$ of the CNN-ATM model is higher than 98% for both training and test sets, which is significantly better than the modeling results of the other methods. The results show that the method can achieve accurate classification for the objective mission compared with traditional machine learning methods because the CNN-ATM network has a powerful feature learning capability to achieve sufficient mining of feature information. However, the high redundancy of hyperspectral data and the drawback of large data volume lead to the difficulty of processing hyperspectral data directly, reducing the computational speed and robustness of the model. Therefore, it is necessary to select feature wavelengths to remove redundant information to obtain a subset of wavelengths with rich information and low correlation.

#### 3.3.2. Detection Results Based on Feature Wavelength

To better evaluate the effectiveness and importance of the proposed CNN-FES network for feature wavelength selection, we compare the performance of hyperspectral data input models after feature selection, and Table 3 shows the classification results for LDA, RF, SVM, and CNN-ATM models. The results show that CNN-FES does not increase the training time, but improves the accuracy and efficiency of the classification task to some extent. Overall, the MSC preprocessing method has slightly inferior modeling results than the DE, and the feature wavelength selection algorithm CNN-FES differs less from SPA and CARS. After analyzing the experimental results, it can be seen that when the values of $A_{C}$, $S_{E}$, and $S_{P}$ are close or equal, the number of sample subsets selected by CNN-FES is less or equal to that of SPA and CARS, and are concentrated at the peak of the spectral curve. The high accuracy obtained from the model indicates that the wavelength variables selected by the method are chemically significant to some extent.

Compared to the results based on full-wavelength modeling, the results of RF and SVM decreased on average by 3.33% and 2.50%, respectively. This may be due to the relatively small differences in spectral characteristics of some samples, resulting in a slight decrease in the detection accuracy. However, the accuracy of all models is above 90% on both the training and test sets, and their discriminatory ability is LDA > SVM > CNN-ATM > RF in descending order, with the highest accuracy of 97.50% for CNN-ATM. It indicates that the performance of the spectral data does not degrade significantly after feature selection, which proves the effectiveness and rationality of the CNN-FES feature selection method in this study.

CNN-ATM reduces overfitting and improves generalization by exploiting attention mechanisms to improve the expressiveness of spectral features with the learning ability of the model. It also utilizes the joint action of multiple convolutional layers to reduce the complexity of the model while retaining key feature information. Moreover, when the model performance was evaluated, the excellent $S_{E}$ (98.28%) and $S_{P}$ (96.77%) values proved the high accuracy of CNN-ATM on the classification task. Although CNN-ATM has an average of 7 s more running time than the machine learning model, it can reach a steady state in a shorter time. This indicates that the proposed model is more robust and has more potential for achieving end-to-end problem-solving.

Although researchers have combined CNN with hyperspectral techniques to detect maize seed quality, fewer studies have addressed the identification of maize seed defects. Detailed comparisons between related literature are difficult due to the differences in crops, methods, and disciplines. However, when considering the use of the same detection method (spectroscopic techniques), experimental object (agricultural products), and agricultural task (defect detection). This study presents a rough comparison of the proposed method with related studies on similar problems. As shown in Table 4, this comparison considered five main aspects: agricultural product type, device type, sample size, spectral range, and accuracy. It can be seen that the accuracy rates for all agricultural tasks are above 83.00%. Among them, the experimental results of this paper (97.50%) are better than the related studies in Table 4. This indicates that the method proposed in this study performs better and enables accurate identification of defective maize seeds.

In summary, CNN-FES and CNN-ATM proposed in this study can simultaneously perform spectral feature wavelength selection and sample classification and achieve good performance and results. These results indicate that 1D-CNN is a feasible method for hyperspectral data analysis, which is of great help to the detection and application of defective maize seeds. Therefore, the CNN model of feature selection and classification proposed by us is of great significance in practical production, which can be applied to hyperspectral data sets for other agricultural tasks, such as disease detection and abiotic stress response. In addition, as the number of wavelengths decreases, portable spectral imaging devices can be developed to improve data acquisition efficiency for practical applications. At present, the detection technology of hyperspectral is developing toward online applications and industrial production. In future work, it is necessary to deeply optimize the network architecture to maintain the accuracy and robustness of the model. At the same time, the variety and number of maize seed samples should be increased to provide accurate quantitative analysis. Based on this, image feature information and spectral feature information can be fused to improve the performance of the wavelength selection algorithm and the efficiency of defect detection.

## 4. Conclusions

This study successfully detected defective maize seeds using HSI combined with deep learning. DE and MSC algorithms were used to pre-process the raw spectra and highlight the spectral differences between different classes. We propose a novel CNN-FES-based feature selection method to select effective feature wavelengths from spectral data. The results show that the number of selected features is fewer than SPA and CARS, and it has better representativeness, interpretability, and classification performance. In addition, a CNN-ATM model based on the attention classification mechanism was proposed for detecting the presence of defects in maize seeds and compared with LDA, RF, and SVM models. The results show that the classification accuracy based on full-wavelength modeling is 98.21%, which is not significantly different from the classification performance of machine learning methods, and the attention mechanism has an excellent ability to emphasize the valid information of the spectral. It demonstrates the feasibility of using hyperspectral data combined with CNN for defect detection in maize seeds, even though these samples have similar shapes and colors. In addition, the modeling accuracy based on the characteristic wavelength variables also reached 97.50%. This indicates that the choice of feature wavelength in the spectral analysis is an effective way to achieve a simplified model. Therefore, the optimal wavelength can be set in advance when using a portable spectral camera to collect sample spectral data, which can effectively improve the processing speed of the model and its applicability to industrial production. In addition, the data obtained were statistically analyzed and compared with related research works in this field. In conclusion, the 1D-CNN proposed in this study is a promising method for hyperspectral wavelength selection and analysis with the potential for online detection, which can provide a reference for the seed industry to improve seed quality.

## Figures and Tables

**Table 1:** Feature wavelengths selected by different algorithms.

Method	Algorithm (Number)	Feature Wavelengths (nm)
DE	SPA (23)	911, 922, 925, 935, 959, 970, 980, 987, 1000, 1142, 1183, 1345, 1397, 1413, 1436, 1657, 1682, 1685, 1689, 1692, 1695, 1698, 1701
	CARS (34)	956, 1256, 1259, 1262, 1269, 1272, 1276, 1282, 1286, 1292, 1295, 1299, 1302, 1305, 1309, 1312, 1315, 1318, 1322, 1328, 1355, 1358, 1361, 1364, 1368, 1371, 1374, 1378, 1381, 1423, 1619, 1644, 239, 1666
	CNN-FES (24)	1239, 1243, 1249, 1252, 1256, 1259, 1262, 1266, 1269, 1272, 1276, 1279, 1282, 1286, 1289, 1292, 1295, 1299, 1302, 1305, 1312, 1368, 1381, 1407
MSC	SPA (24)	897, 911, 915, 918, 922, 946, 959, 963, 966, 970, 1000, 1048, 1176, 1183, 1246, 1358, 1397, 1413, 1436, 1666, 1682, 1685, 1689, 1695
	CARS (29)	980, 990, 1017, 1024, 1028, 1031, 1045, 1243, 1266, 1269, 1276, 1279, 1282, 1289, 1305, 1315, 1361, 1364, 1371, 1374, 1378, 1384, 1420, 1436, 1439, 1443, 1578, 1619, 1632
	CNN-FES (24)	897, 911, 915, 918, 922, 946, 959, 963, 966, 970, 1000, 1048, 1176, 1183, 1246, 1358, 1397, 1413, 1436, 1666, 1682, 1685, 1689, 1695

**Table 2:** Detection results based on full wavelength.

Method	Model	$A_{C}(\%)$		$S_{E}(\%)$		$S_{P}(\%)$		Time (s)
		Train	Test	Train	Test	Train	Test	
DE	LDA	100	97.50	100	96.55	100	98.39	1.36
	RF	98.93	93.33	97.93	89.06	100	98.21	1.34
	SVM	98.21	96.67	97.24	93.55	99.26	100	1.38
	CNN-ATM	98.21	98.33	98.59	100	97.83	96.67	11.73
MSC	LDA	100	95.00	100	93.33	100	96.67	1.37
	RF	99.29	91.67	98.61	87.50	100	96.43	1.36
	SVM	95.00	95.83	92.11	93.44	98.44	98.31	1.35
	CNN-ATM	97.86	98.21	98.59	100	96.38	98.39	12.18

**Table 3:** Detection results based on feature wavelength.

Method	Feature Select	Model	$A_{C}(\%)$		$S_{E}(\%)$		$S_{P}(\%)$		Time (s)
			Train	Test	Train	Test	Train	Test	
DE	SPA	LDA	99.28	100	100	100	98.55	100	1.38
		RF	97.14	92.50	99.30	100	94.93	85.48	1.37
		SVM	98.57	98.33	98.59	100	98.55	96.77	1.38
		CNN-ATM	98.57	96.67	97.18	100	97.10	93.55	8.38
	CARS	LDA	98.93	100	100	100	97.83	100	1.36
		RF	100	92.50	100	100	98.28	87.10	1.37
		SVM	98.57	99.17	98.59	100	98.55	98.39	1.35
		CNN-ATM	92.14	95.00	89.44	94.83	94.93	95.16	9.55
	CNN-FES	LDA	99.64	100	100	100	99.28	100	1.37
		RF	100	91.67	100	91.38	100	89.06	1.35
		SVM	95.36	97.50	95.07	100	95.65	95.16	1.34
		CNN-ATM	93.57	97.48	98.55	100	91.30	93.55	8.36
MSC	SPA	LDA	97.50	99.17	100	100	94.93	98.39	1.34
		RF	98.93	89.17	99.30	93.10	98.55	85.48	1.38
		SVM	96.43	97.50	99.30	100	93.48	95.16	1.38
		CNN-ATM	94.29	92.50	98.59	96.55	89.86	88.71	9.17
	CARS	LDA	98.21	99.17	100	100	96.38	98.39	1.37
		RF	98.93	92.50	100	96.55	97.83	88.71	1.36
		SVM	97.50	98.33	99.30	95.65	100	96.77	1.34
		CNN-ATM	97.50	97.50	96.48	98.28	98.55	96.77	8.86
	CNN-FES	LDA	98.93	99.17	100	100	97.83	98.39	1.36
		RF	100	90.00	100	93.10	100	87.10	1.36
		SVM	91.79	94.17	94.37	94.83	89.13	93.55	1.37
		CNN-ATM	98.21	97.50	100	98.28	96.38	96.77	8.50

**Table 4:** Comparison of the proposed method with related studies.

Agricultural Product Type	Device Type	Sample Size	Spectral Range	Accuracy	References
Sugar beet seed	Terahertz time-domain spectroscopy	100	0.25–0.35 THz	87.00%	[48]
Maize kernel	Multispectral imaging	910	375–970 nm	83.00%	[49]
Wheat kernel	Terahertz time-domain spectroscopy	240	0.1–3.5 THz	96.00%	[50]
Cowpea seed	Raman spectroscopy	105	400–1800 nm	93.70%	[51]
Maize kernel	Hyperspectral imaging	240	953–2517 nm	93.30%	[52]
Maize seed	Hyperspectral imaging	400	900–1700 nm	97.50%	This study

## Data Availability

The data that support the findings of this study are available upon request from the authors.
